# Localized Epithelioid Peritoneal Mesothelioma: A Rare Mimicker of Gynecological Malignancy

**DOI:** 10.7759/cureus.112299

**Published:** 2026-07-08

**Authors:** Francisco Carvalho, Daniela Pereira, Teresa Margarida Cunha

**Affiliations:** 1 Radiology, Unidade Local de Saúde de Gaia e Espinho, Vila Nova de Gaia, PRT; 2 Pathology, Instituto Português de Oncologia de Lisboa Francisco Gentil, Lisbon, PRT; 3 Radiology, Instituto Português de Oncologia de Lisboa Francisco Gentil, Lisbon, PRT

**Keywords:** bap1, immunohistochemistry, localized epithelioid peritoneal mesothelioma, magnetic resonance imaging, pelvic mass

## Abstract

Localized epithelioid peritoneal mesothelioma (LEPM) is a rare variant of peritoneal mesothelioma that presents as a solitary, circumscribed mass rather than with the diffuse serosal spread that characterizes the far more common diffuse form. Because it may arise in the pelvis and be accompanied by ascites and a mildly elevated carbohydrate antigen (CA)-125 level, it is readily mistaken for a gynecological or primary peritoneal malignancy. Although it is generally regarded as less aggressive than the diffuse form, its clinical behavior can be unpredictable. We report the case of a 76-year-old woman in whom a 5 cm pelvic mass was detected incidentally on transvaginal ultrasound and was initially suspected to be a gynecological malignancy.

Preoperative MRI demonstrated clear cleavage planes between the mass and the uterus and left ovary, indicating a non-gynecological origin, and surgery confirmed the presence of a tumor on the antimesenteric border of the small bowel. Histopathology, supported by a targeted immunohistochemical panel (showing calretinin, WT1, D2-40, and CK7 positivity and loss of BAP1), established the diagnosis of LEPM. Despite an apparently localized presentation and complete resection, early peritoneal recurrence developed on follow-up imaging, prompting adjuvant systemic therapy and cytoreductive surgery (CRS). This report underscores that LEPM should be considered in the differential diagnosis of a solitary pelvic mass, highlights the importance of cross-sectional imaging combined with a specific immunohistochemical panel for distinguishing it from gynecological tumors and gastrointestinal stromal tumor (GIST), and emphasizes the need for close follow-up even when the disease appears localized.

## Introduction

Mesothelioma is a rare neoplasm arising from mesothelial cells that line the serous membranes. The pleura is the most commonly affected site, whereas the peritoneum is the second most frequent location, accounting for approximately 10-15% of all mesotheliomas, equivalent to only a few hundred new cases annually in the United States [1,2]. Unlike pleural mesothelioma, which predominantly affects men, peritoneal mesothelioma occurs in men and women with a more balanced sex distribution and typically presents in middle-aged and older adults. Asbestos exposure remains the most well-established risk factor, although its association with the peritoneal form is less pronounced than that observed in pleural mesothelioma; in addition, prior therapeutic abdominal or pelvic radiation has also been implicated [1,2].

Peritoneal mesothelioma typically presents as diffuse disease, characterized by widespread nodular or plaque-like peritoneal involvement and ascites. In contrast, the localized variant - a solitary, sharply circumscribed tumor that is histologically identical to the diffuse form but lacks serosal dissemination - is exceptionally rare and has been reported only in isolated cases [3,4,5]. Its clinical and imaging features closely resemble those of ovarian and primary peritoneal serous carcinoma, and these entities can be reliably distinguished only through a targeted immunohistochemical panel [6].

We present this case report to raise awareness of localized epithelioid peritoneal mesothelioma (LEPM) as an uncommon and often under-recognized cause of a solitary pelvic mass and to demonstrate how preoperative MRI combined with a focused immunohistochemical panel can distinguish it from gynecological malignancies. This distinction is clinically important, as accurate diagnosis may significantly influence prognostic assessment and therapeutic decision-making.

## Case presentation

In February 2026, a 76-year-old woman was referred to our institution after a pelvic mass was incidentally identified during a routine transvaginal ultrasound. She was asymptomatic, reporting no abdominal pain, distension, altered bowel habits, urinary symptoms, weight loss, or anorexia. Her medical history included hypertrophic cardiomyopathy, arterial hypertension, and hypercholesterolemia, in addition to a previous right spontaneous pneumothorax. Her only prior surgery was an appendectomy, with no history of other surgical procedures or hospital admissions. There was no known history of asbestos exposure.

On clinical examination, a well-defined, firm, mobile, and painless pelvic mass was palpable, without evidence of peripheral lymphadenopathy. Laboratory evaluation revealed a mild elevation in carbohydrate antigen (CA) 125 (36.2 U/mL), while carcinoembryonic antigen (CEA) and CA 19-9 levels were within normal limits. Colonoscopy, upper gastrointestinal endoscopy, and cervical cytology showed no evidence of malignancy. In this older woman with a solid pelvic mass, ascites, and mildly elevated CA 125, the leading differential diagnoses included ovarian or primary peritoneal carcinoma; however, a gastrointestinal stromal tumor (GIST) and other mesenchymal or peritoneal neoplasms were also considered.

**Figure 1:**
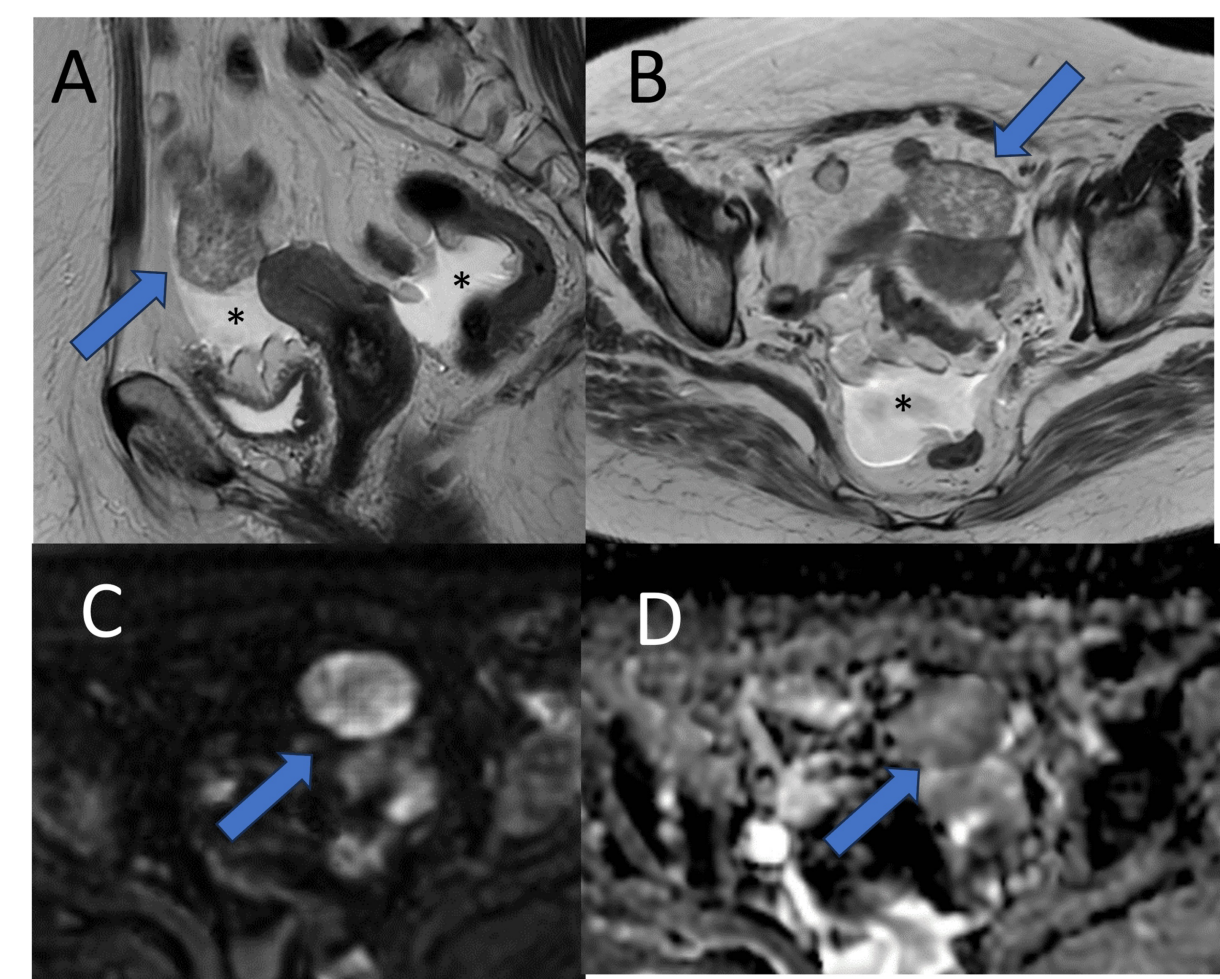
Preoperative MRI (A) Sagittal and (B) axial T2-weighted images demonstrating a well-circumscribed, heterogeneously hyperintense T2 mass (arrows) measuring 5 cm. Clear cleavage planes are identified between the mass and the gynecological organs. Mild pelvic ascites is also present (asterisks). (C) Axial diffusion-weighted image (b-value 1000) and (D) corresponding ADC map of the pelvis. The mass demonstrates restricted diffusion, characterized by high DWI signal intensity and corresponding low ADC signal, suggesting high tissue cellularity MRI: magnetic resonance imaging; ADC: apparent diffusion coefficient; DWI: diffusion-weighted imaging

Because of the initial suspicion of a gynecological malignancy, pelvic MRI was performed. It revealed a 5 cm left paramedian anterior pelvic mass that was isointense on T1-weighted images and heterogeneously hyperintense on T2-weighted images; no intravenous contrast was administered. The lesion demonstrated restricted diffusion, with high signal on high-b-value diffusion-weighted imaging (b = 1000 s/mm²) and correspondingly low apparent diffusion coefficient (ADC) values, findings consistent with high tissue cellularity. Critically, clear cleavage planes were identified between the mass and both the uterine fundus and the left ovary, arguing against an adnexal or uterine origin; moderate pelvic ascites was also present (Figure 1).

In March 2026, the patient was scheduled for cytoreductive surgery (CRS) and underwent a median laparotomy. Intraoperatively, a small amount of serous ascites was noted, together with a 5 cm vegetating tumor arising from the antimesenteric border of the small bowel; there was no macroscopic disease on the parietal peritoneum or omentum. A complete tumorectomy was performed, and omental biopsies were obtained, which were negative for tumor.

**Figure 2:**
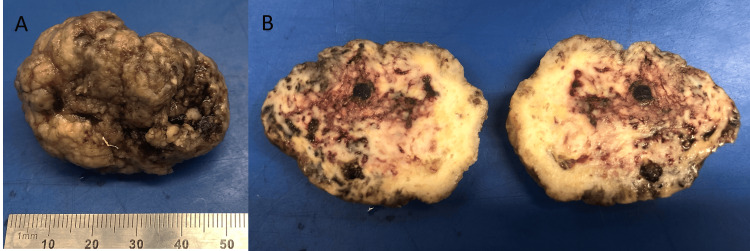
Macroscopic appearance of the resected tumor (A) Gross examination revealed a 5.0 x 4.1 x 3.0 cm tumor with an irregular surface. (B) The cut section demonstrated a firm, whitish parenchyma characterized by hemorrhagic zones and small cystic areas

**Figure 3:**
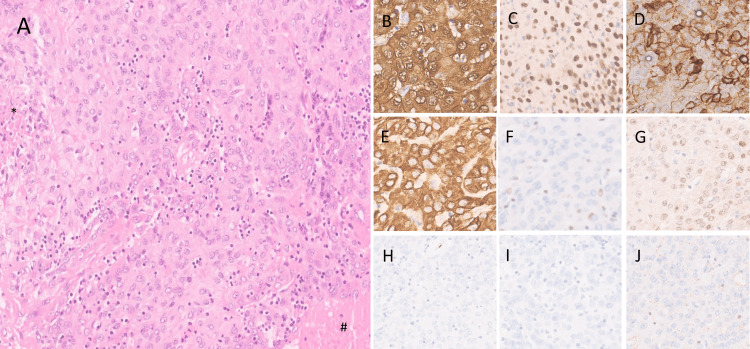
Histological examination of the tumor Solid epithelioid neoplasia with necrosis (*) and hemorrhagic foci (#). The neoplastic cells have abundant cytoplasm, mild nuclear atypia, and there is permeation by lymphocytes (A). Immunohistochemical profile: expression of calretinin (B), WT1 (C), D2-40 (D) and CK7 (E); loss of expression of BAP1 (F); faint and heterogeneous expression of PAX8 (G); no expression of SOX17 (H), CD117 (I) and DOG1 (J)

Gross examination showed a 5.0 × 4.1 × 3.0 cm mass with an irregular surface; the cut surface was firm and whitish, with hemorrhagic zones and small cystic areas (Figure 2). Histology revealed a low-grade epithelioid neoplasm characterized by abundant cytoplasm, mild nuclear atypia, a low mitotic count (1 mitosis/2 mm²), lymphocytic permeation, and areas of necrosis. Immunohistochemistry demonstrated expression of calretinin, WT1, D2-40, and CK7, along with loss of BAP1, confirming the diagnosis of epithelioid mesothelioma; PAX8 was weakly and heterogeneously expressed, whereas SOX17, CD117, and DOG1 were negative (Figure 3).

The postoperative course was uneventful, with no complications. Given the apparently localized nature of the disease and complete macroscopic resection, no adjuvant therapy was administered initially, and the patient was managed with resection alone and close imaging surveillance. However, a follow-up CT performed in June 2026, approximately three months after surgery, demonstrated two new peritoneal implants, with the larger measuring 2 cm, consistent with early peritoneal recurrence. In view of this finding, a multidisciplinary decision was made to proceed with adjuvant systemic therapy and CRS.

## Discussion

This case of LEPM represented a diagnostic pitfall because its presentation, including a solitary pelvic mass, ascites, and a mildly elevated CA-125, closely overlaps with ovarian and primary peritoneal serous carcinoma. This mimicry is well documented, and reliable differentiation of peritoneal mesothelioma from ovarian and peritoneal serous carcinoma depends on a targeted immunohistochemical panel rather than morphology alone [6]. The same entity can also be mistaken for other neoplasms depending on its anatomical location: in a recently reported case, a 76-year-old patient with localized epithelioid peritoneal mesothelioma adjacent to the hepatic flexure was clinically interpreted as having a GIST and was correctly reclassified as mesothelioma only after immunohistochemical analysis, following a work-up analogous to ours [5]. Furthermore, in our case, preoperative MRI excluded an adnexal or uterine source before surgery by demonstrating intact cleavage planes, whereas in several published cases, the distinction was only resolved on final histopathological examination [7,8]. This supports a broader role for the radiologist within the multidisciplinary team, using the cleavage-plane sign to redirect suspicion away from the gynecological organs.

A second topic relates to tumor extent and its implications for treatment. Diffuse peritoneal mesothelioma typically demonstrates widespread peritoneal dissemination and is managed with aggressive CRS and hyperthermic intraperitoneal chemotherapy (HIPEC), whereas the rarer localized variant lacks dedicated treatment guidelines [1,9]. In reported cases of localized epithelioid disease, however, outcomes have varied: in one case, resection without HIPEC was followed by prolonged disease-free survival [5], whereas in another, early peritoneal recurrence developed after resection alone and subsequently required CRS and HIPEC [3]. Our patient followed this latter course: she was initially managed with resection alone, as there was no macroscopic peritoneal disease and the omental biopsies were negative; however, early peritoneal recurrence on follow-up imaging prompted a transition to adjuvant systemic therapy and CRS. Together, these observations illustrate that an apparently localized radiological and intraoperative presentation does not guarantee localized biological behavior, that distinguishing truly localized disease from early or occult diffuse disease can be challenging, and that close postoperative surveillance is therefore essential [5].

The diagnosis of epithelioid mesothelioma rests on characteristic hematoxylin-eosin morphology supported by a robust immunohistochemical panel; the combination of calretinin, WT1, and D2-40 expression with loss of BAP1, as seen here, is highly supportive [10]. The principal challenge in a pelvic mass is separation from a Müllerian carcinoma, complicated by the fact that a subset of epithelioid mesotheliomas express PAX8, the marker traditionally used to denote gynecological origin [11]. In our case, PAX8 was only faintly and heterogeneously positive, whereas SOX17 - a more recently described marker with greater specificity for ovarian and endometrial carcinoma that is characteristically negative in mesothelial proliferations - was negative, reinforcing the mesothelial diagnosis [12]. Finally, because the tumor arose from the antimesenteric border of the small bowel, GIST was an important consideration; negativity for CD117 and DOG1 excluded it [13].

## Conclusions

We described the case of a 76-year-old woman in whom a solitary pelvic mass was detected incidentally and initially attributed to a gynecological malignancy. Preoperative MRI showed intact cleavage planes between the mass and the gynecological organs, correctly redirecting the diagnostic consideration toward an extra-genital peritoneal/intestinal process. A focused immunohistochemical panel, demonstrating positivity for calretinin, WT1, and D2-40 with loss of BAP1, together with negativity for SOX17, CD117, and DOG1, established LEPM while excluding gynecological carcinoma and GIST. The case-specific lessons are threefold: LEPM should be included in the differential diagnosis of a solitary pelvic mass; the cleavage-plane sign on MRI serves as a practical indicator of the organ of origin; and, even when disease appears localized and is completely resected, early peritoneal recurrence can occur. Therefore, close imaging surveillance is essential, and treatment may need to be escalated to systemic therapy and CRS.
